# Supplementary figures and images for: Transcriptomic Analysis of Drug-Resistance Acinetobacter baumannii under the Stress Condition Caused by Litsea cubeba L. Essential Oil via RNA Sequencing
Source: Genes (Basel). 2021 Jun 29;12(7):1003. doi: 10.3390/genes12071003 (PMC8307839; doi:10.3390/genes12071003)

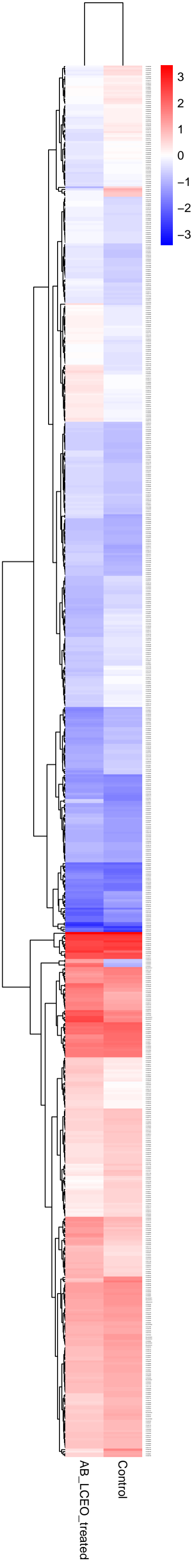

Supplement: Supplementary file 1 [file genes-12-01003-s001.zip › Supplementary Figure S1 Heatmap.pdf]
